# Supplementary material for: Amino acid competition shapes Acinetobacter baumannii gut carriage
Source: bioRxiv. 2024 Oct 19:2024.10.19.619093. Preprint. [Version 1] doi: 10.1101/2024.10.19.619093 (PMC11537318; doi:10.1101/2024.10.19.619093)
Supplement: Supplement 1 [file media-1.pdf]

Supplemental information

Amino acid competition shapes *Acinetobacter baumannii* gut carriage

Xiaomei Ren[1,†], R. Mason Clark[1,†], Dziedzom A. Bansah[1,#], Elizabeth N. Varner[2], Connor R. Tiffany[3], Kanchan Jaswal[1], John H. Geary[1], Olivia A. Todd[1], Jonathan D. Winkelman[4], Elliot S. Friedman[5], Babette S. Zemel[6,7], Gary D. Wu[5], Joseph P. Zackular[3,8,9], William H. DePas[2], Judith Behnsen[1], Lauren D. Palmer[1,*]

[1]Department of Microbiology and Immunology, University of Illinois Chicago, Chicago, IL, USA

[2]Department of Pediatrics, University of Pittsburgh School of Medicine, Pittsburgh, PA, USA

[3]Division of Protective Immunity, Children's Hospital of Philadelphia, Philadelphia, Pennsylvania, USA

[4]Trestle LLC, Milwaukee, WI, USA

[5]Division of Gastroenterology and Hepatology, Perelman School of Medicine, University of Pennsylvania, Philadelphia, Pennsylvania, USA

[6]Department of Pediatrics, Perelman School of Medicine University of Pennsylvania, Philadelphia, Pennsylvania, USA

[7]Division of Gastroenterology, Hepatology, and Nutrition, Children's Hospital of Philadelphia, Philadelphia, Pennsylvania, USA

[8]Department of Pathology and Laboratory Medicine, Perelman School of Medicine, University of Pennsylvania, Philadelphia, Pennsylvania, USA

[9]Center for Microbial Medicine, Children's Hospital of Philadelphia, Philadelphia, Pennsylvania, USA

[†]Contributed equally
[*]Corresponding author
Lauren D. Palmer
835 S Wolcott Ave
MSB E703
Chicago, IL 60612
ldpalmer@uic.edu
[#]Present address: American University of the Caribbean, Cupecoy, Sint Maarten

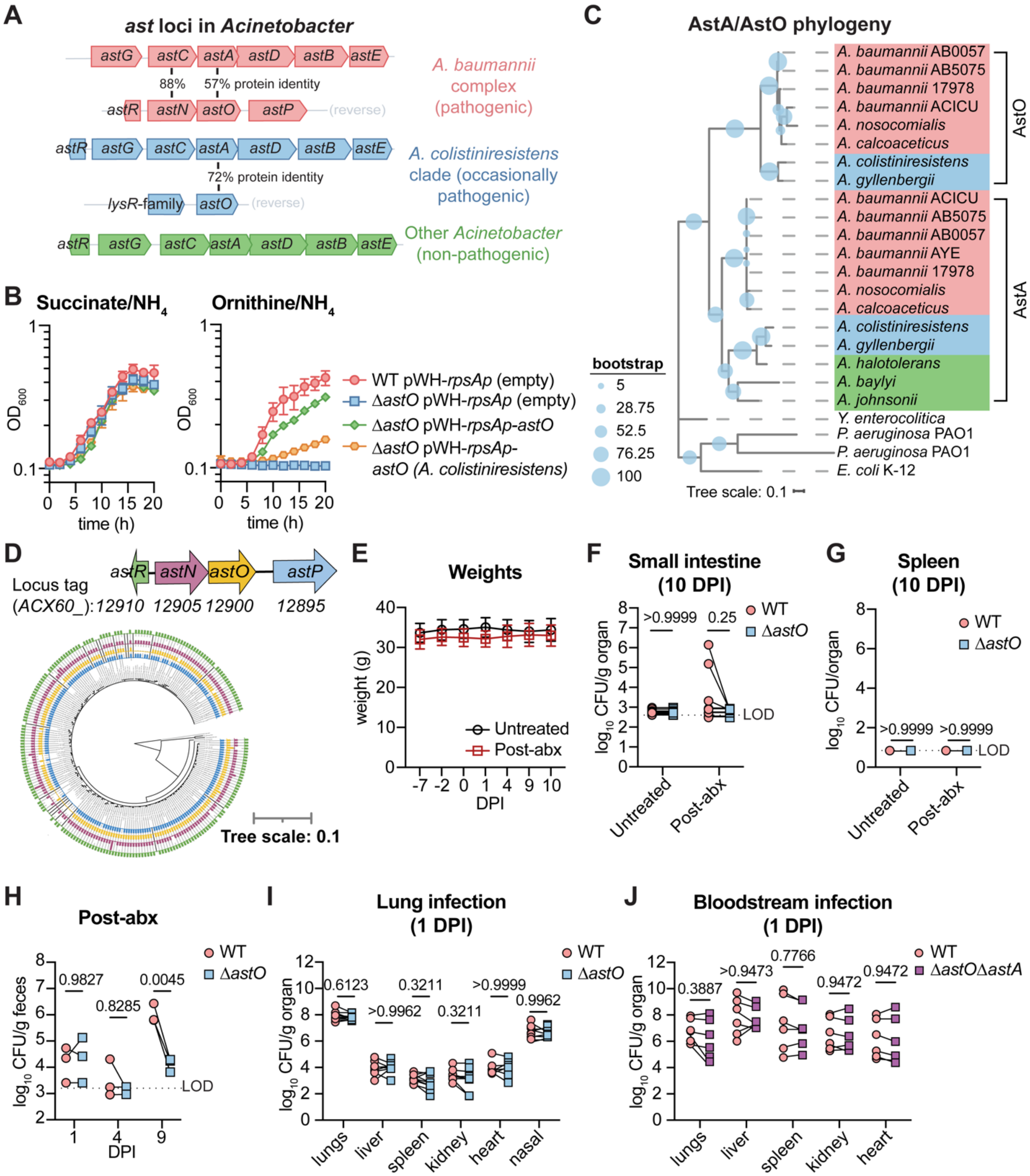

Fig S1. Evidence of evolution at the second *ast* locus in *Acinetobacter* spp. and requirement for *A. baumannii* gut colonization.

Corresponds with Figure 1

(A) *ast* loci in *Acinetobacter* clades. Members of the *abc* clade such as *A. baumannii* 17978 encode the second *ast* locus with *astR*, and *astNOP* on the negative strand (red). Members of the *A. colistiniresistens* clade encode the *astGCADBE* locus with *astR* divergently transcribed; the second *ast* locus has only *astO* and a divergent LysR-family regulator gene (blue). Other non-pathogenic *Acinetobacter* spp. only one *ast* locus with *astR* and *astGCABDE* (green).

(B) Growth of *A. baumannii* 17978 WT pWH (empty vector), Δ*astO* pWH, Δ*astO* pWH-*astO* (*A. baumannii*) and Δ*astO* pWH-*astO* (*A. colistiniresistens*) grown in M9 minimal media with succinate or ornithine as the sole carbon source. Growth was monitored by OD_{600} measurement for 20 h (n = 3, mean +/- SD).
(C) Phylogenetic tree of AstA and AstO proteins (tree scale in amino acid substitutions).
(D) Second *ast* locus genes and their corresponding copy numbers mapped to an *A. baumannii* species phylogenetic tree generated from 233 de-duplicated published *A. baumannii* and *Acinetobacter* genomes (see Table S2; tree scale in amino acid substitutions).
(E) Weight of female Swiss Webster mice in Figure 1G-H (n = 10, mice combined from 2 independent experiments, mean ± SD).
(F) *A. baumannii* 17978 CFU in the small intestine at 10 DPI from mice shown in Figure 1G-H (n = 10, mice combined from 2 independent experiments, *p* by Wilcoxon test with Holm-Sidak's multiple comparisons).
(G) *A. baumannii* 17978 CFU in the spleen at 10 DPI from female Swiss Webster mice shown in Figure 1G-H (n = 10, mice combined from 2 independent experiments; *p* by Wilcoxon test with Holm-Sidak's multiple comparisons).
(H) Male Swiss Webster mice were administered gentamicin as in Figure 1F and at 0 DPI were inoculated with 1:1 *A. baumannii* 17978 WT and Δ*astO*. CFU were enumerated from feces at 1, 4, and 9 DPI (n = 3, *p* by two-way ANOVA with Sidak's multiple comparisons).
(I) Female C57BL/6 mice were intranasally inoculated with 1:1 *A. baumannii* 17978 WT and Δ*astO*. CFU were enumerated at 1 day post inoculation (DPI) (n = 10, *p* by Wilcoxon test with Holm-Sidak's multiple comparisons).
(J) Male C57BL/6 mice were retroorbitally inoculated with 1:1 *A. baumannii* 17978 WT and Δ*astA*Δ*astO*. CFU were enumerated at 1 DPI (n = 6, *p* by Wilcoxon test with Holm-Sidak's multiple comparisons).
DPI, days post inoculation; LOD, average limit of detection.

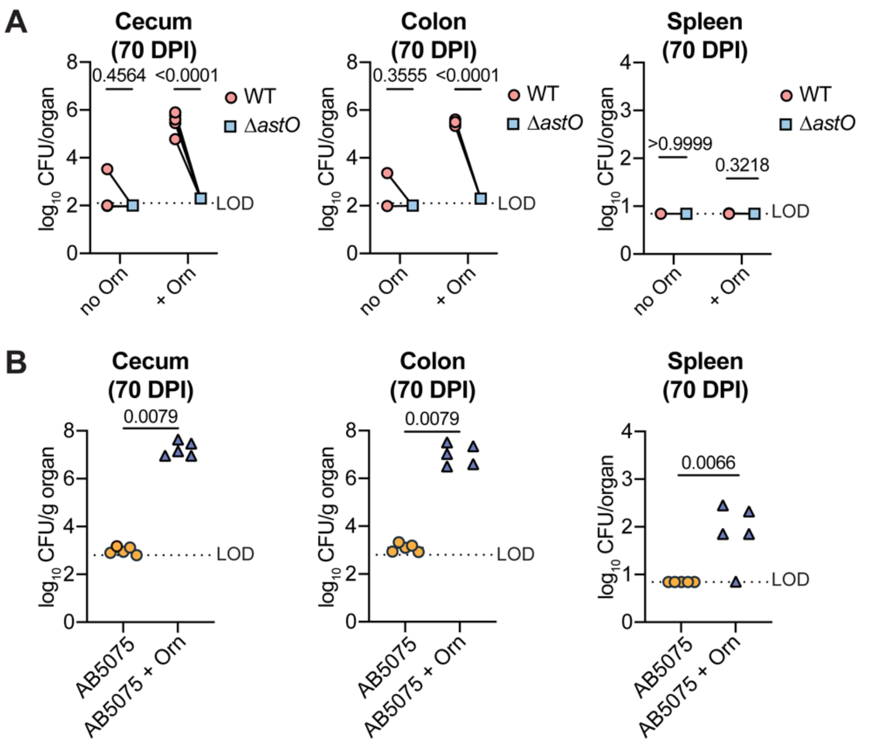

Figure S2 Supplemental ornithine promotes long-term *A. bauma*nnii gut colonization

Corresponds with Figure 2

(A) Female C57BL/6J mice (from Figure 2B) were euthanized at 70 DPI and *A. baumannii* CFU were enumerated from the cecum, colon and spleen (n = 5, *p* by two-way ANOVA with Sidak's multiple comparisons).

(B) Female Swiss Webster mice (from Figure 2D) were euthanized at 70 DPI and *A. baumannii* 5075 CFU were enumerated from the cecum, colon and spleen (n = 5, *p* by two-way ANOVA with Sidak's multiple comparisons).

DPI, days post infection; LOD, average limit of detection.

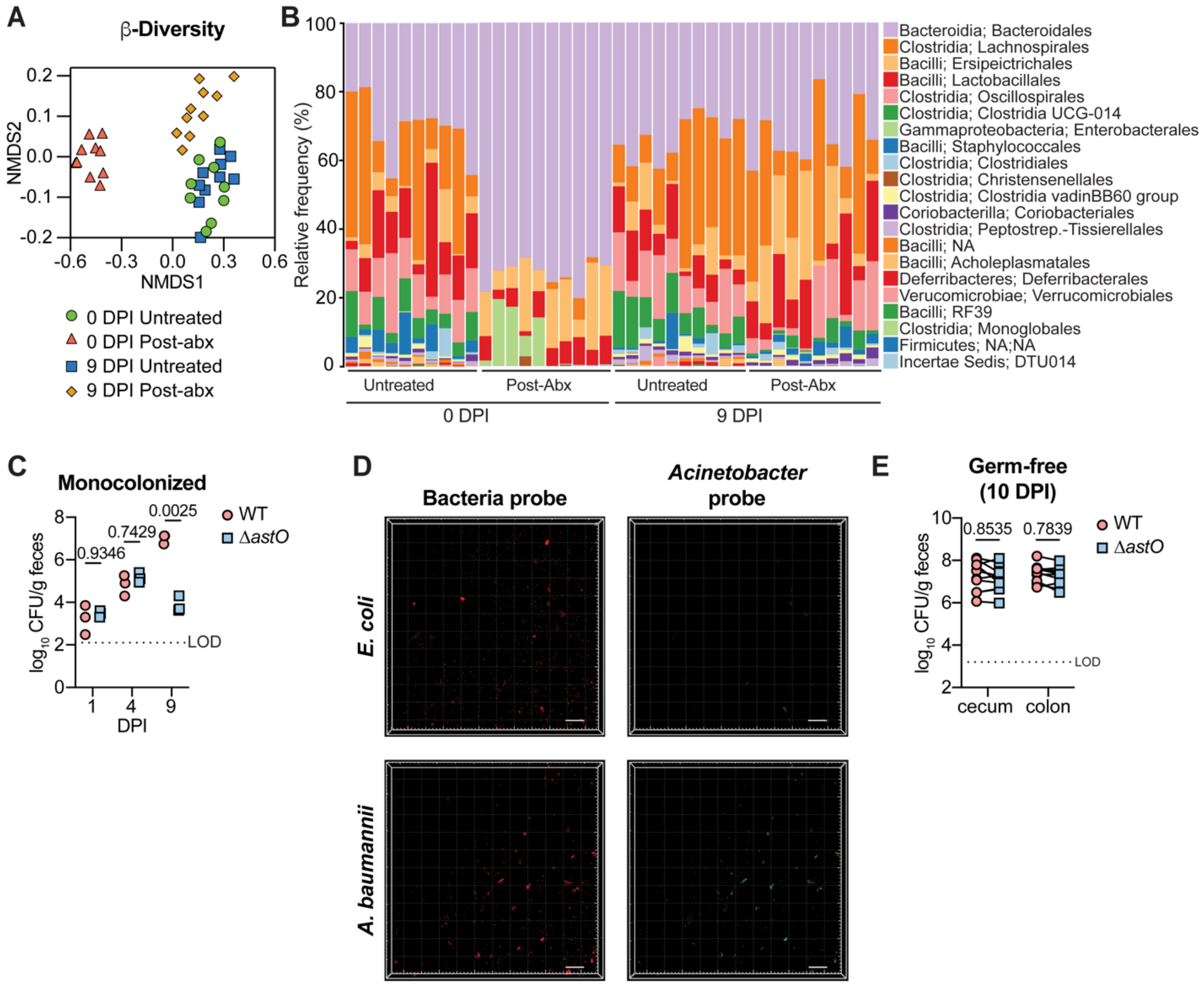

Figure S3 WT *A. baumannii* outcompetes Δ*astO* by 9 DPI.

Corresponds with Figure 3

(A) β-diversity NMDS plot of 16S rRNA gene profiling at 0 and 9 DPI in the feces of untreated and post-abx female Swiss Webster mice inoculated with *A. baumannii* 17978 WT and Δ*astO* and shown in Figure 1G-H (n = 10).

(B) Relative abundance of bacterial ASV identified by 16S rRNA gene sequencing at 0 and 9 DPI in the feces of untreated and post-abx female Swiss Webster mice shown in Figure 1G-H (n = 10).

(C) Mice were mono-inoculated with *A. baumannii* 17978 WT or Δ*astO*. CFU were enumerated from fecal samples at 1, 4 and 9 DPI (n = 3 female Swiss Webster mice, *p* by two-way ANOVA with Sidak's multiple comparisons). Mice inoculated with WT were used for MiPACT-HCR imaging shown in Figure 4B.

(D) MiPACT-HCR imaging of bacterial cultures to assess specificity of anti-*Acinetobacter* probe Aci16s 729. Scale bar is 50 µm; green, anti-*Acinetobacter* HCR probe; red, general bacterial HCR probe eub338.

(E) GF mice were euthanized at 10 DPI and CFU were enumerated (n = 10, *p* by two-way ANOVA with Sidak's multiple comparisons).

NMDS, non-metric multidimensional scaling; DPI, days post infection; ASV, amplicon sequence variants; LOD, limit of detection; MiPACT-HCR, microbial identification after passive clarity technique via hybridization chain reaction.

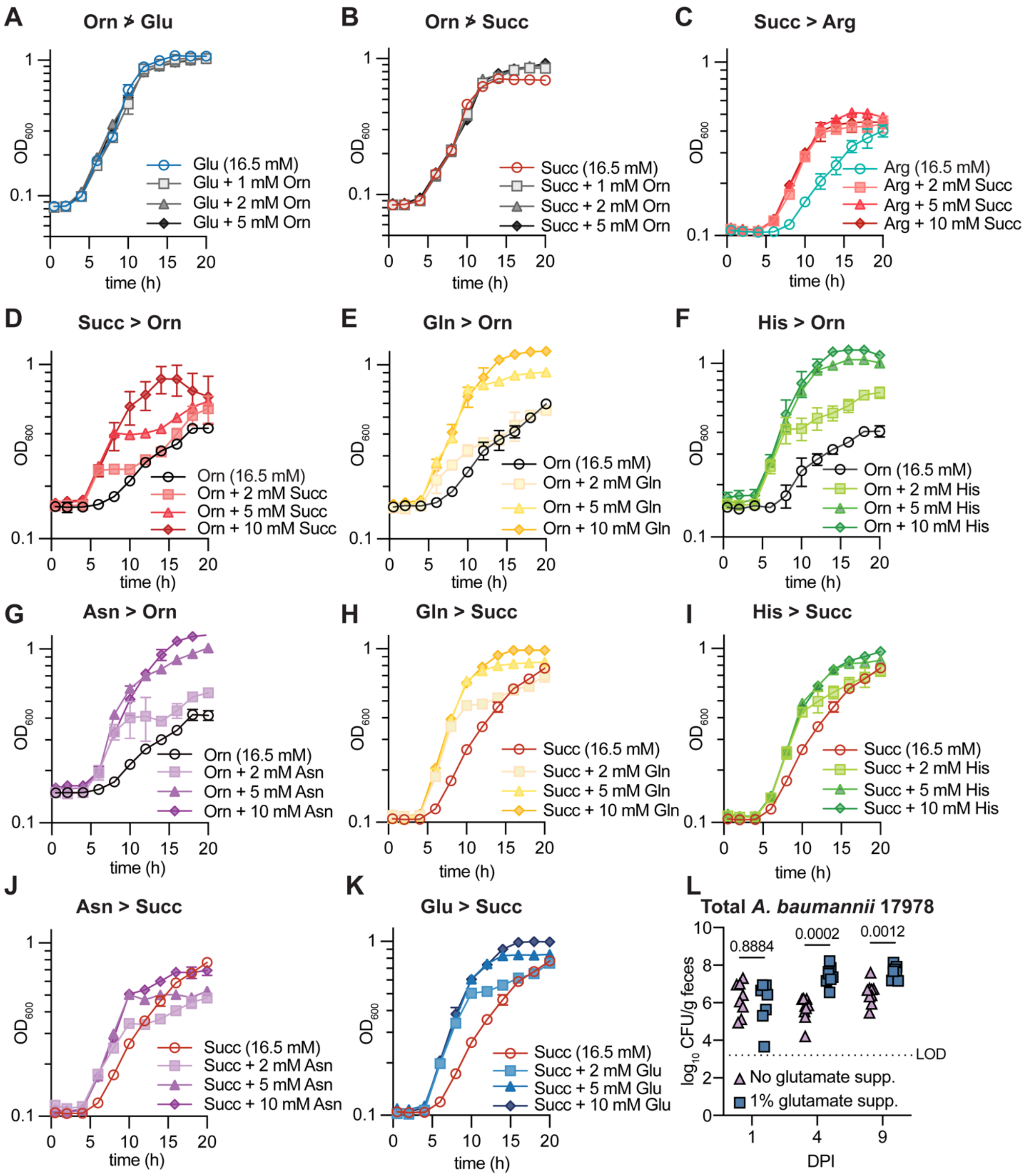

Figure S4 ***A. baumannii*** **preferred carbon source**

Corresponds with Figure 4

(A-K) *A. baumannii* 17978 WT growth curve in M9 media containing 16.5 mM carbon source and indicated additions as the sole carbon sources. Growth was monitored by OD_{600} (n = 3, mean ± SD). *A. baumannii* preferred carbon source is Glu, Gln, Asn, His > Succ > Arg, Orn

(L) Post-abx mice were co-inoculated with *A. baumannii* 17978 WT and Δ*astO* and one group was supplemented with 1% monosodium glutamate in the drinking water. Total *A. baumannii* CFU were enumerated from fecal samples at 1, 4 and 9 DPI (n = 8-9 female Swiss Webster mice also shown in Figure 4E-F, *p* by two-way ANOVA with Sidak's multiple comparisons). Orn, ornithine; Glu, glutamate; Succ, succinate; Gln, glutamine; His, histidine; Asn, asparagine; Arg, arginine. DPI, days post infection; LOD, limit of detection.

Table S1. Strains, plasmids, and oligonucleotides.

REAGENT or RESOURCE	SOURCE	IDENTIFIER
Bacterial and virus strains		
Acinetobacter baumannii ATCC 17978VU	ATCC (Wijers et al., 2021)	LP486
Acinetobacter baumannii ATCC 17978VU*::mTn7*	This study	LP526
A. baumannii Δ*astO* deletion strain used in IN infection (old one)	This study	LP720
A. baumannii Δ*astO* Δ*astA* deletion strain used in RO infection	This study	LP516
Acinetobacter baumannii ATCC 17978VU Δ*astO::kan*	This study	LP814
Acinetobacter baumannii ATCC 17978VU *astA1*$^{L125A\ H229A}$	This study	LP838
Acinetobacter baumannii ATCC 17978VU *astA1*$^{L125A\ H229A}$Δ*astO::kan*	This study	LP872
Acinetobacter baumannii ATCC 17978VU pWH1266-P_{rpsA}	This study	LP731
Acinetobacter baumannii ATCC 17978VU Δ*astO::kan* pWH1266-P_{rpsA}	This study	LP732
Acinetobacter baumannii ATCC 17978VU Δ*astO::kan* pWH1266-P_{rpsA}-*astO*	This study	LP1019
Acinetobacter baumannii ATCC 17978VU Δ*astO::kan* pWH1266-P_{rpsA}-*astO(A. colistiniresistens)*	This study	LP1020
Acinetobacter baumannii ATCC AYE	ATCC	LP13
Acinetobacter baumannii 0057	Robert Bonomo (Case Western Reserve University)	LP14
Acinetobacter baumannii ACICU	M. Stephen Trent (University of Georgia)	LP293
Acinetobacter baumannii ABUW AB5075	Colin Manoil (University of Washington)	LP345
Acinetobacter nosocomialis	Mario Feldman (Washington University at St. Louis)	LP116
Acinetobacter baylyi ADP1	ATCC	LP459
Acinetobacter colistiniresistens	DSMZ	LP697
Acinetobacter gyllenbergii	DSMZ	LP698
Escherichia coli K12	Maria Hadjifrangiskou (Vanderbilt University Medical Center)	LP235
Escherichia coli BW25113	Matthew Chapman (University of Michigan)	BW25113
Pseudomonas aeruginosa	Andrea Battistoni (University of Rome Tor Vergata)	LP346
Oligonucleotides		
gttaaaaaggatcgatcctctagaggatcCTATACAAATGAACCCGTTCTAC		*astO*_up_F
agctccagcctacacGCTGCTTGTTCATCCTTTTG		*astO*_up_R
gaggatattcatatgGCGGAAATTACTATACATTTCAC		*astO*_dn_F
tgaccatgattacgaattcgagctcggtacCATTTGGAGAAGTAAACCC		*astO*_dn_R
GTGTAGGCTGGAGCTGCTTC		pKD4-FRTfrag_F
CATATGAATATCCTCCTTAGTTCCTATTC		pKD4-FRTfrag_R
GTTAAAAAGGATCGATCCTCTAGAatgtacttacgactgcaaaag		*astA1*_up_F
aaTGCtgtacagagctcactac		*astA1*_L125A_R
agtgagctctgtacaGCAttttta		*astA1*_L125A_F
tgtggTGCcatttttccaatca		*astA1*_H229A_R
aaaaatgGCAccacatactttgcc		*astA1*_H229A_F
AATTCGAGCTCGGTACCtacaactgtgttaccagcaagt		*astA1*_dn_F
TTATCAGGTATATCctcgagatgatgattattcgttacattgaac		Com_*astO*_F
GGGCATCGGTCGACggtaccattaacagccattcgaaaat		Com_*astO*_R
TTATCAGGTATATCctcgagATGATGCTGATTCGTTATATCA		Com_*astO(A.colistiniresistens)*_F
GGGCATCGGTCGACggtaccTCAATTTTTCTTTGGAATATTG		Com_*astO(A.colistiniresistens)*_R

gttaaaaaggatcgatcctctagaggatcCTATACAAATGAACCCGTTCTAC		*astO*_up_F
agctccagcctacacGCTGCTTGTTCATCCTTTTG		*astO*_up_R
gaggatattcatatgGCGGAAATTACTATACATTTCAC		*astO*_dn_F
tgaccatgattacgaattcgagctcggtacCATTTGGAGAAGTAAACCC		*astO*_dn_R
GTGTAGGCTGGAGCTGCTTC		pKD4-FRTfrag_F
CATATGAATATCCTCCTTAGTTCCTATTC		pKD4-FRTfrag_R
AGAGTTTGATYMTGGCTCAG		16s-CS1_27F-YM
AGAATTTGATCTTGGTTCAG		16s-CS1_27F-Chl
AGAGTTTGATCCTGGCTTAG		16s-CS1_27F-Bor
AGGGTTCGATTCTGGCTCAG		16-CS1_27F-Bif
AGAGTTCGATCCTGGCTCAG		16s- CS1_27F-Ato
ATTACCGCGGC GCTGG		534R
Recombinant DNA		
pFLP2	Hoang et al., 1998	N/A
pKD4	Datsenko and Wanner, 2000	N/A
pKNOCK-mTn7-Amp	Carruthers *et al.*, 2013	N/A
pWH1266	Hunger *et al.*, 1990	N/A
pWH1266-*rpsAp*	Palmer *et al.*, 2020	pLDP29
pWH1266-*rpsAp-astO*	This study	pLDP171
pWH1266-*rpsAp-astO (colistiniresistens)*	This study	pLDP181
pFLP2-Δ*astO::Kn*	This study	pLDP94
pFLP2-*astA1*$^{L125A\ H229A}$	This study	pLDP216
